# Surgery for brain metastases—impact of the extent of resection

**DOI:** 10.1007/s00701-021-05104-7

**Published:** 2022-01-26

**Authors:** Rebecca Rootwelt Winther, Marianne Jensen Hjermstad, Eva Skovlund, Nina Aass, Eirik Helseth, Stein Kaasa, Olav Erich Yri, Einar Osland Vik-Mo

**Affiliations:** 1 European Palliative Care Research Centre (PRC), Department of Oncology, Oslo University Hospital, and Institute of Clinical Medicine, University of Oslo, Oslo, Norway; 2 Norwegian University of Science and Technology, Trondheim, Norway; 3 Institute of Clinical Medicine, University of Oslo, Oslo, Norway; 4 Department of Neurosurgery, OUH, Oslo, Norway

**Keywords:** Brain metastases, Surgery, Extent of resection, Survival

## Abstract

**Background:**

Surgical resection of brain metastases improves symptoms and survival in selected patients. The benefit of gross total resection is disputed, as most patients are believed to succumb from their non-CNS tumor burden. We investigated the association between overall survival and residual tumor after surgery for single brain metastases.

**Methods:**

We reviewed adults who underwent surgery for a single brain metastasis at a regional referral center (2011–2018). Gross total resection was defined as no visible residual tumor on cerebral MRI 12–48 h postoperatively.

**Results:**

We included 373 patients. The most common primary tumors were lung cancer (36%) and melanoma (24%). We identified gross total resection in 238 patients (64%). Median overall survival was 11.0 months, 8.0 (6.2–9.8) months for patients with subtotal resection and 13.0 (9.7–16.3) months for patients with gross total resection. In a multivariate regression analysis including preoperative prognostic factors, gross total resection was associated with longer overall survival (HR: 0.66, *p* = 0.003). Postoperative radiotherapy administered within 6 weeks did not significantly alter the hazard ratio estimates for grade of resection.

**Conclusions:**

Our study suggests improved survival with gross total resection compared to subtotal resection. The importance of extent of resection in surgery for brain metastases should not be discarded.

## Introduction

Metastases to the brain are the most common malignant brain tumors in adults and result in high morbidity and mortality [1]. Patients experience debilitating symptoms, often including neurological deficits and psychological distress [5, 17, 26]. Median overall survival varies across diagnostic groups, general oncological status, and number and size of the metastasis, but is generally short: around 5 months after diagnosis [3]. The most common primary cancers seen in patients with brain metastases are lung, melanoma, breast, and colorectal cancer [16]. Therapeutic strategies differ between patients, aiming at symptom control; life prolongation; and, in rare cases, curation. Possible treatment options include radiotherapy, systemic medical therapy, or surgery. Radiotherapy can be given as whole brain radiotherapy (WBRT) or stereotactic radiotherapy (SRT), depending on the number and size of the lesions, as well as the general oncological status of the patients. Systemic medical therapy has historically not been very useful in brain metastases, but in the last decade, immunotherapy and targeted therapies have induced promising research, especially in patients with brain metastases from melanoma and non-small cell lung cancer [10, 12, 20]. A combination of treatment modalities is often used [22].

Surgery is generally preferred in patients with a limited number of intracerebral lesions, lesions with a total volume exceeding the limit for SRT, in cases where mass effect or edema is symptomatic or resulting in hydrocephalus, or where histopathological diagnosis is uncertain [2, 14]. Surgery may relieve symptoms and prolong survival [22]. Gross total resection is attempted whenever possible, but difficulties in identifying tumor margins and fear of inflicting damage to eloquent structures may result in unintended subtotal resection. The intraoperative evaluation of tumor borders can be challenging. Surgeons may overestimate the extent of resection in their intraoperative assessment, resulting in a discrepancy with postoperative MRI in up to 40% of the cases [19]. Therefore, postoperative MRI within 72 h is used to determine the extent of resection [7].

In gliomas, more extensive tumor resection is associated with longer overall survival [8]. In single brain metastases on the other hand, a recent study by Jünger et al. found that the extent of resection did not influence overall survival in a group of 197 patients who received adjuvant treatment (postoperative radiotherapy and systemic therapy) [6]. However, other studies report both longer overall survival and longer time to local recurrence in patients with confirmed gross total resection compared to subtotal resection in brain metastases [7, 11, 19]. An increased focus on gross total resection may result in more patients developing postsurgical neurological damage. Thus, the impact of gross total resection of brain metastases needs further investigation. We examined the median overall survival in patients with gross total resection vs. subtotal resection of single-brain metastases to establish further the clinical importance of grade of resection after surgical resection of brain metastases.

## Methods

### Patients

We reviewed the electronic medical records of all 374 adults who underwent surgical resection of a single-brain metastasis from a solid tumor in the time period 2011–2018 at Oslo University Hospital (OUH), identified through the hospital’s Brain Tumor Register. OUH is the only regional referral center for neurosurgery in the South-Eastern Norway Health Region, part of a public single-payer healthcare system, with a population of 3 million: 55% of the Norwegian population. Exclusion criteria were more than one brain metastasis or leptomeningeal dissemination at the time of surgery, age under 18 years, or no available postoperative MRI imaging. Last follow-up was June 2, 2021.

### Classification of preoperative variables

Eastern Cooperative Oncology Group (ECOG) performance status was retrieved from electronic patient records the last week prior to surgery. In cases where no ECOG status was noted, estimation was done from written descriptions the last days prior to surgery. In case of hemiparesis and/or reduced consciousness due to intracranial metastasis, the ECOG status was set to 4 (patient confined to bed). Status of extracranial disease was categorized as (1) stable: no documented new metastases or growing primary tumor within the last 3 months prior to brain metastasis surgery, (2) progressive: growing primary tumor/metastases or new metastases within the last 3 months prior to surgery, (3) synchronous: primary tumor discovered within 1 month prior to surgery or brain metastasis as first sign of disease, or (4) unknown disease status: no radiological staging 3 months prior to surgery, but known primary cancer.

### Surgery

All patients were referred to the Department of Neurosurgery for consideration for surgery by the treating oncologist. Indication for surgery was determined by an experienced specialist in neurosurgery. All included patients underwent craniotomy, and most patients were operated with neuro-navigation and peroperative frozen-section neuropathological evaluation. Four patients were operated with awake mapping and bipolar cortical and subcortical mapping.

### Extent of resection

Gross total resection was defined as no visible residual tumor 12–48 h postoperatively as described by neuroradiologists in the electronic patient records. In cases of ambiguity concerning postoperative tumor remnant on postoperative MRI, patients were classified in the subtotal resection group. Most patients had a T1 contrast-enhanced 3D spin echo series on 1.5 MRI, combined with axial T2 and FLAIR 3D series preoperatively. Some patients treated early in the period had T1 contrast-enhanced series only in three planes and not in a standardized 3D protocol. However, all patients were evaluated by T1 contrast-enhanced 3D spin echo series on 1.5 MRI, combined with axial T2 and FLAIR 3D series postoperatively. The number of brain metastases on pre- and postoperative imaging was double-checked to ensure that there were no patients with more than one brain metastasis at the time of surgery included in the study.

### Classification of postoperative complications and neurological deficits

We included complications related to the surgical resection of brain metastases or intubation/anesthesia within 30 days of surgery for brain metastases as described in electronic patient records. These include severe neurological deterioration, intracranial hemorrhage described on postoperative cerebral imaging, intracerebral abscess, bone flap infection, CSF leakage, and pneumonia. Bone flap infection could occur after the 30-day period. Neurological deficits were registered based on electronic patient records preoperatively and 1–3 days postoperatively. Postoperative neurological deficits were classified as unchanged, better, worse, much worse (severe neurological deterioration or coma), or unknown.

### Statistical analyses

Overall survival was estimated using the Kaplan–Meier estimator and the log-rank test was used to assess differences in overall survival. Patients still alive were censored at last follow-up (June 2021). Hazard ratios were estimated by Cox’ proportional hazards model. The proportionality assumption was checked by visual inspection of log–log plots. Categorical variables were compared between groups by the chi-square test. *P*-values below 0.05 were regarded as statistically significant. Statistical analyses were performed in SPSS Statistics 26 (IBM Corp., Armonk, NY).

Postoperative radiotherapy could be a confounding variable in our dataset. If administered, postoperative radiotherapy was given within 6 weeks after surgery, but at varying time points, resulting in the risk of immortal time bias. To avoid this bias, we performed landmark survival analyses [13] starting 6 weeks after surgery. We ran two of these survival analyses, one with and one without postoperative radiotherapy, thereby investigating the influence of postoperative radiotherapy on the hazard ratio estimates of extent of resection.

## Results

### Patient characteristics

Of 374 patients, one was excluded due to missing postoperative imaging. Of the remaining 373 patients, 52% were female and median age was 63 (range 18–89) years at the time of surgery. The most common primary tumors were lung (36%) and melanoma (24%), while 11% had unknown origin. None of the patients had small cell lung cancer. Gross total resection was confirmed on postoperative cerebral MRI in 239 patients (64%). The overall complication rate was 7%; the most common complications were intracranial hemorrhage (3%), CSF leakage (1%), and pneumonia (1%). In total, 228 patients (61%) received postoperative radiotherapy within 6 weeks after surgery. Fifty eight (16%) patients received SRT, and 166 (44%) received WBRT or partial brain radiotherapy (PBRT).

### Differences by extent of resection

Distribution of patient characteristics and postoperative variables in the gross total resection and subtotal resection groups is shown in Tables 1 and 2. The proportion of patients with poor functioning status (high ECOG score) was higher in the subtotal resection group (*p* < 0.001). There was no significant difference in postoperative complications within 30 days after surgery between patients with gross total and subtotal resection (*p* = 0.61) (Table 2).

**Table 1:** Patient characteristics

Variables	Total *N* *N* (%)	Gross total resection *N* (%)	Subtotal resection *N* (%)
Number of patients	373	238 (64)	135 (36)
Median age	63 (range: 18–89)	64	63
Gender			
Female	195 (52)	123 (52)	72 (53)
Male	178 (48)	115 (48)	63 (47)
Preoperative ECOG performance status			
0	57 (15)	41 (17)	16 (12)
1	145 (39)	103 (43)	42 (31)
2	107 (29)	65 (27)	42 (31)
3–4	64 (17)	29 (12)	35 (26)
Primary tumor			
Lung	134 (36)	79 (33)	55 (41)
Melanoma	89 (24)	59 (25)	30 (22)
Colorectal	39 (11)	27 (11)	12 (9)
Breast	26 (7)	22 (9)	4 (3)
Kidney	19 (5)	13 (5)	6 (4)
Other	39 (11)	26 (12)	13 (10)
Unknown origin	27 (7)	12 (5)	15 (11)
Extracranial metastases			
Yes	182 (49)	117 (49)	65 (48)
No	172 (46)	113 (47)	59 (44)
Unknown	19 (5)	8 (3)	11 (8)
Status of extracranial disease^a^			
Stable	115 (31)	81 (34)	34 (25)
Progressive	62 (17)	41 (17)	21 (16)
Synchronous	125 (34)	75 (32)	50 (37)
Unknown disease status	71 (19)	41 (17)	30 (22)
Location of brain metastasis			
Infratentorial	90 (24)	59 (25)	31 (23)
Supratentorial	283 (76)	179 (75)	104 (77)
Median size (largest diameter)	38 mm	37 mm	40 mm

^a^Stable: no documented new metastases or growing primary tumor in any imaging modality within the last 3 months prior to brain metastasis surgery. Progressive: growing primary tumor/metastases or new metastases 3 months prior to brain metastasis surgery. Synchronous: primary tumor discovered within 1 month prior to brain metastasis surgery/brain metastasis as first sign of disease. Unknown disease status: known primary cancer, but no radiological staging 3 months prior to brain metastasis surgery

**Table 2:** Postoperative variables (after surgery for brain metastases)

Variables	Total *N* *N* (%)	Gross total resection *N* (%)	Subtotal resection *N* (%)
Complications to surgery			
None	346 (93)	222 (93)	124 (92)
Intracranial hemorrhage	10 (3)	7 (3)	3 (2)
Pneumonia or pulmonary embolism	4 (1)	1 (< 1)	3 (2)
Bone flap infection	1 (< 1)	1 (< 1)	0 (0)
CSF leakage	5 (1)	2 (2)	3 (2)
Intracerebral abscess	2 (< 1)	2 (2)	0 (0)
Other complication in need of neurosurgical intervention	5 (1)	3 (1)	2 (1)
Postoperative neurological deficits			
Unknown	68 (18)	46 (19)	22 (16)
Unchanged	215 (58)	142 (60)	73 (54)
Better	64 (17)	36 (15)	28 (21)
Worse	24 (7)	12 (5)	12 (9)
Much worse^a^	2 (< 1)	2 (2)	0 (0)
Postoperative radiotherapy (within 6 weeks after surgery)			
None	114 (31)	72 (30)	42 (31)
SRT	58 (16)	31 (13)	27 (20)
WBRT	147 (39)	102 (43)	45 (33)
PBRT	19 (5)	13 (5)	6 (4)
Both SRT and WBRT	4 (1)	3 (1)	1 (< 1)
Unknown	31 (8)	17 (7)	14 (10)

^a^Severe neurological deterioration

### Survival

**Fig. 1:**
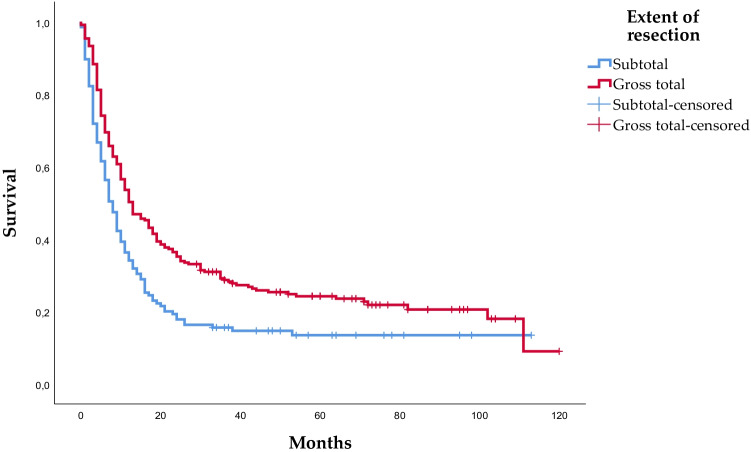
Overall survival in months by extent of resection

Median overall survival was 11.0 months; 8.0 months in the subtotal group and 13.0 months in the gross resection group (*p* < 0.001)**,** illustrated in Fig. 1. When adjusting for known preoperative prognostic factors, general characteristics at baseline and extent of resection (perioperative variables), gross total resection was associated with longer overall survival compared to subtotal resection (HR: 0.663) (Table 3). To investigate the association between postoperative radiotherapy and overall survival and reduce the risk of immortal time bias, we conducted two landmark analyses starting from 6 weeks after surgery for brain metastases. Fourteen patients died within 6 weeks after surgery and were not included. The estimates from the landmark analyses were very similar to the primary analyses: HR = 0.639 (CI: 0.484–0.843) without adjustment for postoperative radiotherapy and HR = 0.627 (CI: 0.474–0.831) with adjustment for postoperative radiotherapy. We also performed subgroup survival analyses on patients with lung cancer and melanoma. For patients with lung cancer, hazard ratio estimates for gross total resection compared to subtotal resection were similar to the main analysis (HR = 0.746, CI: 0.470–1.182). For patients with melanoma, gross total resection had a hazard ratio of 0.872 (CI: 0.453–1.677) compared to subtotal resection.

**Table 3:** Association between perioperative variables and overall survival

	Unadjusted^a^		Full model^b^	
	Hazard ratio (95% CI)	*p*-value	Hazard ratio (95% CI)	*p*-value
Gender				
Female (reference)	*1*		1	
Male	*1.27 (1.01–1.60)*	*0.043*	*1.29 (1.03–1.80)*	*0.029*
Age at time of surgery				
< 60 (reference)	1		1	
60–69	1.10 (0.83–1.46)	0.512	1.00 (0.73–1.38)	0.987
≥ 70	*1.86 (1.38–2.50)*	< *0.001*	*1.81 (1.27–2.56)*	*0.001*
ECOG status				
ECOG 0 (reference)	1		1	
ECOG 1	*1.53 (1.04–2.23)*	*0.030*	*1.54 (1.01–2.37)*	*0.046*
ECOG 2	*2.10 (1.42–3.12)*	< *0.001*	*1.72 (1.12–2.66)*	*0.014*
ECOG 3–4	*2.34 (1.52–3.60)*	< *0.001*	*2.46 (1.50–4.03)*	< *0.001*
Primary tumor				
Lung (reference)	1		1	
Colon/rectum	1.45 (1.00**–**2.10)	0.053	1.48 (0.95**–**2.33)	0.086
Melanoma	0.86 (0.63**–**1.16)	0.312	1.04 (0.49**–**1.79)	0.886
Breast	*0.56 (0.34–0.92)*	*0.023*	0.72 (0.70**–**1.53)	0.306
Kidney	0.84 (0.49**–**1.44)	0.532	0.94 (0.49**–**1.79)	0.839
Other	0.86 (0.57**–**1.29)	0.463	0.98 (0.61**–**1.57)	0.916
Unknown origin	1.16 (0.75**–**1.81)	0.509	0.85 (0.48**–**1.51)	0.584
Chemotherapy any time prior to surgery				
No (reference)	1		1	
Yes	1.23 (0.97**–**1.56)	0.095	1.30 (0.86**–**1.93)	0.215
Extracranial metastases				
No (reference)	1		1	
Yes	*1.56 (1.23–1.98)*	< *0.001*	*1.52 (1.10–2.10)*	*0.011*
Not evaluated	1.07 (0.60**–**1.90)	0.817	0.85 (0.42**–**1.71)	0.647
Status of extracranial disease				
Stable (reference)	1		1	
Progressive	*2.08 (1.47–2.94)*	< *0.001*	1.25 (0.80**–**1.95)	0.324
Synchronous disease	1.27 (0.94**–**1.72)	0.114	1.27 (0.85**–**1.89)	0.247
Unknown	*1.49 (1.07–2.09)*	*0.020*	1.14 (0.74**–**1.76)	0.545
Grade of resection				
Subtotal (reference)	1		1	
Gross total	*0.67 (0.53–0.85)*	*0.001*	*0.66 (0.51–0.87)*	*0.003*
Location of brain metastasis				
Supratentorial (reference)	1		1	
Infratentorial	1.09 (0.83**–**1.43)	0.551	1.21 (0.87**–**1.69)	0.260
Median size (diameter)	*1.17 (1.06–1.29)*	*0.002*	*1.15 (1.04–1.28)*	*0.010*

Italic values = statistically significant values

^a^Unadjusted model = univariable regression analysis

^b^Full model = multivariable regression analysis

## Discussion

The main purpose of this large retrospective study was to understand better the importance of grade of resection in patients who undergo surgery for single brain metastasis. In the study population of 373 patients, we found that 64% had gross total resection on postoperative MRIs. This number is similar to the 61.5% gross total resection rate reported by Kamp et al. in 2015 [7] and 62.4% by Jünger et al. in 2021 [6]. However, the actual gross total resection rate in our study could be higher, since postoperative MRIs that were inconclusive for tumor remnant were classified in the subtotal resection group.

Extent of resection was independently associated with overall survival in our study, also after adjusting for known preoperative prognostic variables such as age, primary cancer, presence of extracranial metastases, extracranial disease status, and ECOG performance status. Lee et al. found a similar positive effect on overall survival in patients with gross total resection in their 2013 study [11]. Contrary, Jünger et al. [6] recently found that extent of resection did not influence overall survival. Their study design and patient population is quite similar to the current study. However, there are important dissimilarities in our dataset and the dataset of Jünger et al. Firstly, their sample size is much smaller than in the current study: 197 vs. 373 patients. Furthermore, there are differences in the composition of the primary cancer diagnoses, with a higher rate of melanomas (24% vs. 10.5%) and a lower rate of lung cancer (36% vs. 46.7%) and breast cancer (7% vs. 13.7%) in the current study. Interestingly, breast cancer is the most common cancer among women in Norway [18]. However, patients with breast cancer usually develop brain metastases late in the disease trajectory. They often receive close follow-up, allowing for early detection of smaller lesions, and as such often receive stereotactic radiotherapy. Patients who undergo surgery for brain metastases are more likely to have larger lesions or brain metastases as first sign of the cancer disease [25]. In addition, Jünger et al. included adjuvant systemic therapy as a variable in their survival analysis. Unfortunately, we do not have systematic registration of adjuvant systemic therapy, because many patients received such treatment at their local hospitals. However, Jünger et al. found no significant difference in adjuvant treatment modality between patients undergoing gross total and subtotal resection [6]. Furthermore, Jünger et al. solely included patients who received postoperative radiotherapy. In the current study, we had access to information on postoperative radiotherapy; however, only 61% underwent such treatment in our sample. Thus, one could speculate that the extent of resection is more important for patients who do not receive postoperative radiotherapy. However, our landmark survival analyses revealed that postoperative radiotherapy did not significantly influence the hazard ratio estimates of extent of resection. The different rates of postoperative radiotherapy are therefore unlikely to fully explain the different findings between the two studies.

Increased focus on gross total resection could result in unintended damage to healthy brain tissue and increase the risk of postoperative complications and patients developing postsurgical neurological deficits. For gliomas, it has been demonstrated that surgically induced neurological deficits is detrimental to prognosis [4], as such deficits make further oncological treatment less feasible. It is highly likely that this also pertains to patients with brain metastases. Reassuringly, we found no significant difference in rate of postoperative complications based on the grade of resection, and the overall postoperative complication rate of 7% is comparable to similar studies [21, 23]. Furthermore, we found no sign of gross total resection being associated with a higher risk of worse neurological deficits in our study. We did not have access to formal prospective standardized neurological examinations [15], but information on neurological deficits preoperatively and 1–3 days after surgery was derived from electronic patient records. However, the neurosurgeons pre- and intraoperative assessment may result in a selection bias where a brain metastasis in an eloquent area is less likely be removed with gross total resection than one located in a non-eloquent area [6].

Registration of postoperative complications and neurological deficits is important when we investigate the clinical impact of gross total resection. However, to achieve patient-centered care, future studies should include patient-reported outcome measures to investigate self-reported symptoms and quality of life in these patients. This will help to understand the full clinical impact of extent of resection in patients undergoing surgery for single brain metastasis.

The use of intraoperative fluorescein [9, 24] may improve the identification of unintended residual tumor, making gross total resection more achievable. Combining this with improved intraoperative neuro-navigation, imaging and monitoring might allow supra-marginal resection to reduce neurological death and increase overall survival, without augmenting the risk of postoperative neurological deficits in this patient group.

### Strengths and limitations

The large sample size is an important strength of this study. Only one patient was excluded due to missing postoperative cerebral MRI. Unfortunately, we did not register the exact number of patients who underwent the different modalities of preoperative MRI. However, all MRI modalities were adequate for tumor surgery. The lack of information on adjuvant systemic treatment administered at local hospitals and standardized pre- and postoperative neurological assessments are important limitations. Furthermore, we were unable to include progression free survival as an outcome in the study because we lack systematic MRIs from the local hospitals in the follow-up period. In addition, since the association between survival and multiple potentially prognostic variables is estimated, there is a risk of false-positive findings. Variables other than extent of resection should be regarded as covariates for adjustment, and *p*-values close to the 5% significance level should be interpreted with caution.

## Conclusions

Previous evidence regarding the association between extent of resection of brain metastases and overall survival is equivocal. Our study demonstrates a survival benefit with gross total resection compared to subtotal resection in patients with single brain metastasis, when adjusting for known prognostic preoperative factors and postoperative radiotherapy. Thus, the importance of extent of resection in surgery for brain metastases should not be discarded.
